# Detection of SARS-COV-2 receptor ACE-2 mRNA in thyroid cells: a clue for COVID-19-related subacute thyroiditis

**DOI:** 10.1007/s40618-020-01436-w

**Published:** 2020-10-06

**Authors:** M. Rotondi, F. Coperchini, G. Ricci, M. Denegri, L. Croce, S. T. Ngnitejeu, L. Villani, F. Magri, F. Latrofa, L. Chiovato

**Affiliations:** 1 Laboratory for Endocrine Disruptors, Unit of Internal Medicine and Endocrinology, Istituti Clinici Scientifici Maugeri IRCCS, 27100 Pavia, PV Italy; 2 Department of Internal Medicine and Therapeutics, University of Pavia, Via S. Maugeri 4, 27100 Pavia, PV Italy; 3 Unit of Molecular Cardiology, Istituti Clinici Scientifici Maugeri IRCCS, 27100 Pavia, Italy; 4 Department of General and Minimally Invasive Surgery, Istituti Clinici Scientifici Maugeri IRCCS, 27100 Pavia, PV Italy; 5 Unit of Pathology, Istituti Clinici Scientifici Maugeri IRCCS, 27100 Pavia, PV Italy; 6 Department of Clinical and Experimental Medicine, University of Pisa, 56124 Pisa, PI Italy

**Keywords:** Thyroid, Thyrocytes, SARS-COV-2, COVID-19, ACE-2

## Abstract

**Purpose:**

SARS-COV-2 is a pathogenic agent belonging to the coronavirus family, responsible for the current global world pandemic. Angiotensin-converting enzyme 2 (ACE-2) is the receptor for cellular entry of SARS-CoV-2. ACE-2 is a type I transmembrane metallo-carboxypeptidase involved in the Renin-Angiotensin pathway. By analyzing two independent databases, ACE-2 was identified in several human tissues including the thyroid. Although some cases of COVID-19-related subacute thyroiditis were recently described, direct proof for the expression of the ACE-2 mRNA in thyroid cells is still lacking. Aim of the present study was to investigate by RT-PCR whether the mRNA encoding for ACE-2 is present in human thyroid cells.

**Methods:**

RT-PCR was performed on in vitro ex vivo study on thyroid tissue samples (15 patients undergoing thyroidectomy for benign thyroid nodules) and primary thyroid cell cultures.

**Results:**

The ACE-2 mRNA was detected in all surgical thyroid tissue samples (*n* = 15). Compared with two reporter genes (GAPDH: 0.052 ± 0.0026 Cycles^−1^; β-actin: 0.044 ± 0.0025 Cycles^−1^; ACE-2: 0.035 ± 0.0024 Cycles^−1^), the mean level of transcript expression for ACE-2 mRNA was abundant. The expression of ACE-2 mRNA in follicular cells was confirmed by analyzing primary cultures of thyroid cells, which expressed the ACE-2 mRNA at levels similar to tissues.

**Conclusions:**

The results of the present study demonstrate that the mRNA encoding for the ACE-2 receptor is expressed in thyroid follicular cells, making them a potential target for SARS-COV-2 entry. Future clinical studies in patients with COVID-19 will be required for increase our understanding of the thyroid repercussions of SARS-CoV-2 infection.

## Introduction

SARS-COV-2 is a pathogenic agent, belonging to the coronavirus family, responsible for the current global world pandemic [1]. The clinical picture of COVID-19 disease is in part similar to that previously reported for other coronavirus infections (i.e. SARS and MERS) [2]. In particular, pneumonia-associated symptoms, acute respiratory distress syndrome (ARDS), sepsis and multiple organ failure [3–6] are peculiar manifestations of severe COVID-19 disease. Unfortunately, at present there are no approved therapies for the treatment of COVID-19. Thus, scientists are making a great effort to elucidate SARS-COV-2 to develop possible effective therapeutic strategies while waiting for the development of a vaccine. In this context, great interest stemmed from the demonstration of a cell membrane receptor through which SARS-COV-2 would enter cells of the host tissue. Several studies suggest that SARS-COV-2, similarly to SARS-COV and NL63/HCoV-NL63, utilizes the Angiotensin-converting enzyme 2 (ACE-2) as a cellular entry receptor [7]. ACE-2 is a type I transmembrane metallo-carboxypeptidase which, being involved in the Renin-Angiotensin pathway, is targeted for the treatment of hypertension [8]. ACE-2 maps to chromosome Xp22, spans 39.98 kb of genomic DNA, and contains 20 introns and 18 exons [9]. ACE-2 is mainly located in extracellular regions (cell membrane) or secreted. It is known to be expressed in a variety of different tissues: in the upper and lower human respiratory tract, in the myocardium and in the gastrointestinal mucosa [7, 10], all tissues shown to harbour SARS-COV-2 [10–12]. Hoffman et al., by using several cell lines, demonstrated that the SARS-CoV-2 infection depends on the presence of host cell receptor ACE-2 [13]. In addition, by using a monkey cell line suitable for SARS-CoV-2 replication, Zhou et al. found that treatment with an anti-ACE-2 antibody prevented the cell entry of SARS-COV-2 [14, 15]. Based on these findings, and on the evidence that ACE-2 is expressed by several tissues, it was hypothesized that SARS-COV-2 would infect not only the respiratory tract cells, a main target of COVID-19 disease, but also other tissue cells expressing ACE-2.

The possibility that SARS-CoV-2 could also infect thyroid cells derives from the notion that different virus-like particles are detectable in the follicular epithelium of patients with subacute thyroiditis (SAT) [16] and, most recently, by some independent case reports of SAT related to SARS-CoV-2 [17–22]. The anatomic location of the thyroid, which is contiguous to the upper airways, a main entrance site of corona viruses, further supports the hypothesis that the thyroid could be a target of SARS-CoV-2. As a matter of fact, it was previously reported that a substantial number of patients with Severe Acute Respiratory Syndrome (SARS-CoV) displayed thyroid function abnormalities and disruption of the follicular architecture [23]. From a clinical point of view, the fact that some COVID-19 patients complain of ear pain (i.e. a classical symptom of SAT) would further support the hypothesis that SARS-CoV-2 could infect the thyroid, thus producing a subacute inflammation. Eventually, this pathologic process could be clinically relevant because SAT-related thyrotoxicosis could contribute to the cardiovascular complications observed in COVID-19 patients. In line with this view, Li et al., by analyzing two independent databases, identified the presence of ACE-2 in several human tissues including the thyroid [24]. However, direct proof for the existence of the ACE-2 mRNA in thyroid cells is still lacking. Aim of the present study was to investigate by RT-PCR whether the mRNA encoding for ACE-2 is expressed by thyroid tissue samples and in primary cultures of thyroid cells.

## Materials and methods

### Thyroid tissue samples

Surgical samples of fifteen thyroids were obtained from the disease-free tissue of patients who underwent thyroidectomy for a nodular goiter (12 women and 3 men). All specimens used in the study were snap frozen at surgery and then maintained at − 80 °C. Written informed consent for the study was obtained from all patients.

### Primary cultures of human thyroid cells

To confirm that the amplified genes specifically belonged to follicular thyroid cells, two primary cultures of normal thyrocytes were also investigated. Briefly, surgical specimens of normal human thyroid were obtained from the contralateral disease-free lobe of patients who underwent thyroidectomy for a solitary malignant nodule (*n* = 2). Surgical specimens were minced and then incubated with collagenase type II (Sigma, Saint Louis, MO, USA) 5 mg/ml, in 5 ml of Coon’s F12 medium, for 4 h at 37 °C. Then, 10 ml of Coon’s F12 medium were added, following which, cells were filtered, spun at 1000 × g for 10 min, washed with Coon’s F12 medium, spun again, and finally re-suspended in complete medium containing 5% newborn calf serum and a mixture of six hormones including insulin (5 μg/ml), hydrocortisone (50 μg/ml), transferrin (5 μg/ml), somatostatin (10 ng/ml), gly-his-lysine (10 ng/ml) and bovine TSH (1 mU/ml).

### Real-time PCR

Total RNA was isolated from thyroid specimens using a Total RNA purification kit according to the manufacturer’s instructions (Norgen Biotek, Canada). Genomic DNA was digested using the DNAse enzyme (Norgen Biotek, Canada) at room temperature for 15 min and following the manufacturer’s protocol. Total RNA from samples was reverse transcribed into cDNA using a Sensi Fast c-DNA synthesis kit (Bioline, London, UK), following the manufacturer’s instructions. Real-time PCR was performed using Sensi-Fast SYBR Green Hi-ROX kit (Bioline, London, UK) on a StepOne Plus Applied Biosystems real-time PCR system. Amplification was done under the following conditions: 95 °C for 2 min; followed by 40 cycles of 95 °C, 5 s and 60 °C, 10 s. β-actin and GAPDH were used as endogenous controls. Pre-designed primers targeting human ACE-2 (F: GGGATCAGAGATCGGAAGAAGAAA; R: AGGAGGTCTGAACATCATCAGTG) GAPDH (F: AAATCCCATCACCATCTTCC; R: GGTTCACACCCATGACGAAC) and β-actin (F: TGCGTGACATGAGAAG; R: GCTCGTAGCTTCTCCA) were obtained from Biomers.net GMBH (Soflinger, Germany). Primers of ACE-2 were chosen based on the study by Ma et al. [25]. All samples were run in triplicate. The means of the number of Cycles^−1^ of ACE-2, GAPDH and β-actin genes were compared in all samples. Moreover, the expression of ACE2 in relation to both GAPDH and β-actin was calculated for all the samples.

## Results

### Expression of ACE-2 mRNA in thyroid tissue specimens

**Fig. 1:**
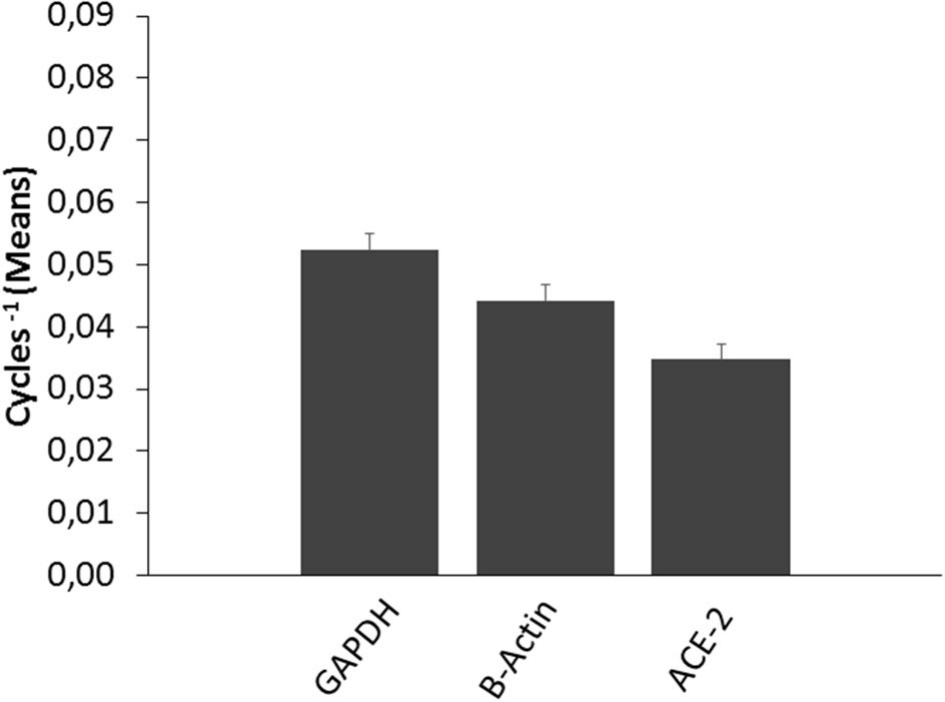
Mean expression of ACE-2 in thyroid tissue specimens. The expression of GAPDH, β-actin and ACE-2 are shown as means of Cycles ^−1^

The expression levels of ACE-2 mRNA were evaluated in 15 different thyroid tissue specimens and in two primary thyroid cells cultures. The ACE-2 mRNA was detected in all thyroid tissue samples. The mean level of transcript expression for ACE-2 mRNA was calculated and compared with that of GAPDH and of β-actin (two reporter genes ubiquitously expressed by cells). As shown in Fig. 1, large amounts of ACE-2 mRNA were detected (GAPDH: 0.052 ± 0.0026 Cycles^−1^; β-actin. 0.044 ± 0.0025 Cycles^−1^; ACE-2: 0.035 ± 0.0024 Cycles^−1^).

**Fig. 2:**
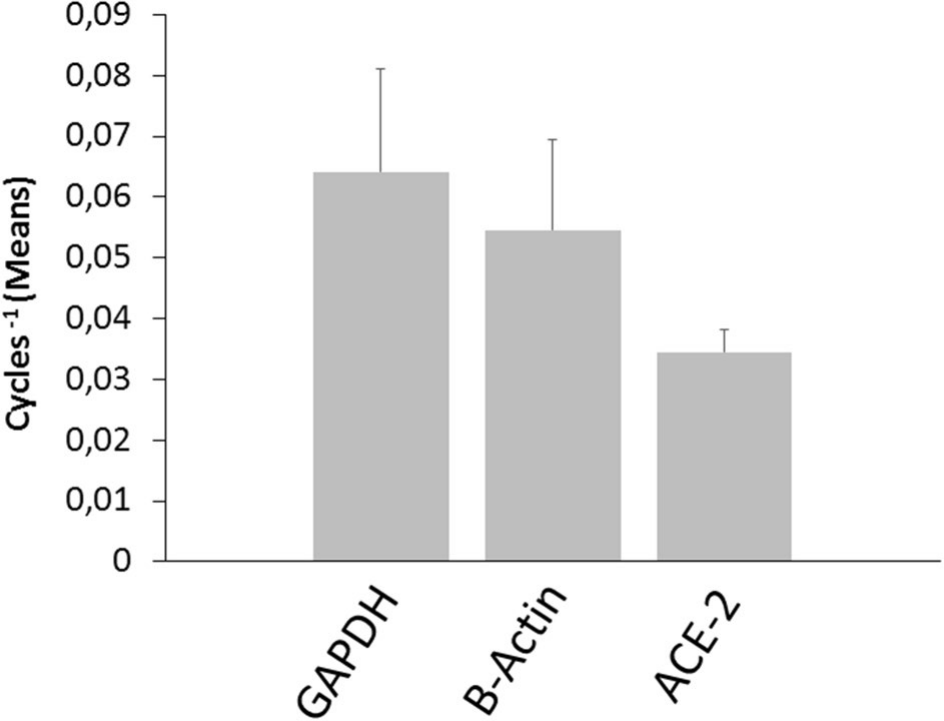
Mean expression of ACE-2 in primary cultures of thyroid cells. The expression of GAPDH, β-actin and ACE-2 are shown as means of Cycles ^−1^

**Fig. 3:**
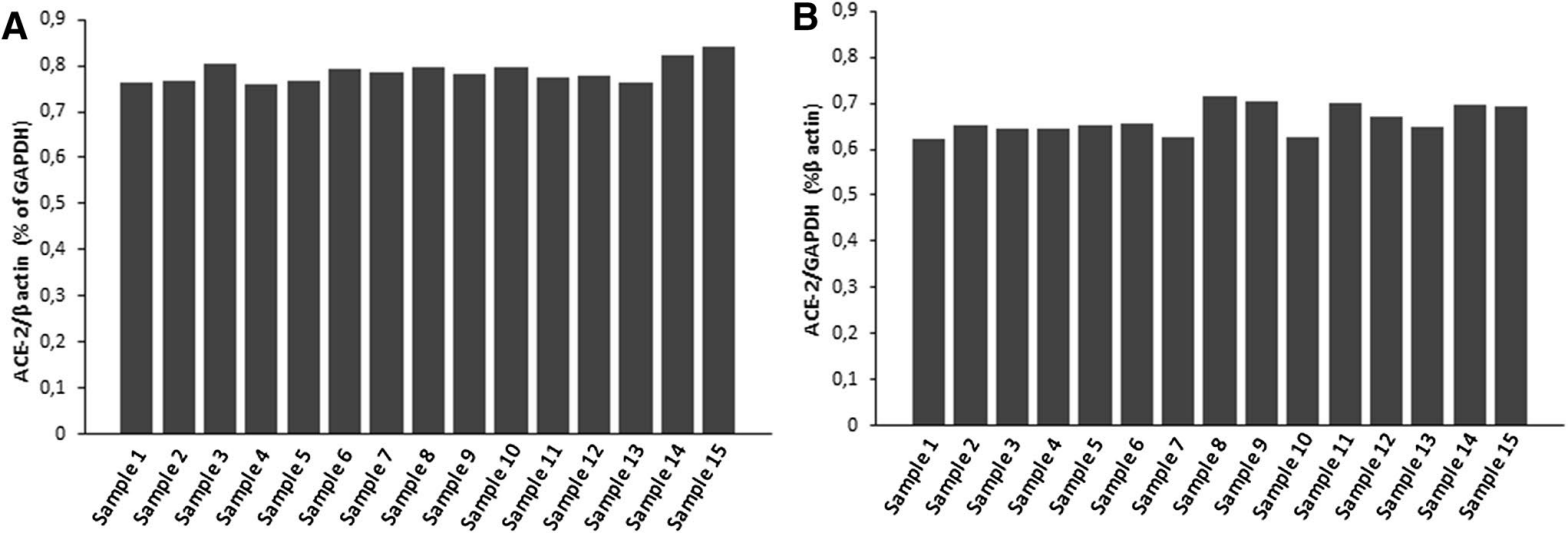
Panel **a** ACE-2/β-actin ratio calculated as percentage of GAPDH. Bars are representative of distinct thyroid tissue specimens. Panel **b** ACE-2/ GAPDH ratio calculated as percentage of β-actin. Bars are representative of distinct thyroid tissue specimens

To confirm the expression of ACE-2 mRNA by follicular cells, primary cultures of thyroid cells were also evaluated (Fig. 2). Interestingly, the expression levels of ACE-2 mRNA were superimposable between thyroid tissue specimens and follicular thyroid cells in primary culture (Figs. 1, 2). These findings would indicate that the total RNA isolated and reverse transcribed into cDNA from tissues derived rather exclusively from follicular thyroid cells. Moreover, the ACE-2/GAPDH mRNAs ratio, related to β-actin, revealed no substantial differences among the 15 thyroid tissue samples (Fig. 3a). The same result was found when the ACE-2/β-actin ratio was calculated as percentage of GAPDH (Fig. 3b). Taken together the above results clearly indicate an overall between-patients homogeneity for the expression levels of the mRNA encoding for the ACE-2 gene.

## Discussion

The present study provides the first demonstration that the mRNA of the SARS-COV-2 receptor, ACE-2 is expressed in thyroid cells. In addition, by comparing the expression levels of ACE-2 mRNA with those of two reporter genes (GAPDH and β-actin), it was observed that the ACE-2 mRNA is abundantly expressed by thyroid cells.

Several studies highlighted that SARS-COV-2 enters the host cells through ACE-2 receptor, a mandatory step for viral replication and transmission [13, 26, 27]. Based on these findings, the expression pattern of ACE-2 mRNA in specific cell types could identify further routes of SARS-CoV-2 infection in humans. Recent studies have shown that SARS-CoV-2 infection not only affects the upper respiratory tract cells, but also involves other organs. Indeed, SARS-CoV-2 was detected in a variety of human tissues belonging to the respiratory, cardiovascular, digestive, urinary and reproductive systems [28, 29]. ACE2 mRNA was found to be expressed in spermatogonia, as well as Leydig and Sertoli cells, thus suggesting that testis may be potentially vulnerable to SARS-CoV-2 infection. Similarly, Jing et al. suggested that SARS-Cov-2 may infect the ovary, uterus, vagina and placenta through the ubiquitous expression of the ACE-2 receptor [30]. ACE-2 expression was also demonstrated in endothelial cells from arterial and venous vessels [31]. More importantly, there is clear-cut evidence that endothelial cells are prone to acquire SARS-CoV-2 infection [32], with subsequent development of endotheliitis, endothelial cell damage, systemic vasculitis and disseminated intravascular coagulation.

To provide evidence on whether the thyroid might be a target organ of SARS-COV-2 infection, the present study aimed at identifying ACE-2 in thyroid cells. RT-PCR analysis showed that the ACE-2 mRNA is expressed at consistent levels in thyroid tissue specimens and cells. These findings are in line with data from previous in silico studies. The detection of the mRNA encoding for ACE-2 in thyroid tissue is for sure a relevant finding. However, immunohistochemical studies will be required to confirm that the ACE-2 protein is present on thyroid cells. The expression level of the ACE-2 mRNA in the thyroid were not compared with that in other organs. However, previous data [33] suggest that expression level of the ACE-2 mRNA in the thyroid, even if relevant, is lower than that in lung, small intestine and colon, and higher than that in breast, liver and skeletal muscle. Further studies, specifically designed to compare the relative expression of the ACE-2 mRNA in different human tissues, will be needed to further clarify this issue.

Taken together, the above described data support the view that the presence of the viral receptor correlates with the susceptibility to SARS-CoV-2 infection and that the receptor expression in tissues other than the lung may explain the multi-organ failure observed in severe cases [34]. As far as the thyroid gland is concerned, Li et al. first reported the expression of ACE-2 in the thyroid, but this was done only by in silico prediction analysis [24]. Our results demonstrate for the first time the expression of ACE2 mRNA in human thyroid surgical specimens and in primary cultures of thyroid cells. It should also be highlighted that, by comparing the expression of ACE-2 mRNA with two different transcripts such as GAPDH and β-actin, the ACE-2 mRNA was found to be abundant and, more importantly, homogenously expressed by thyroid cells.

It is important to note that, as recently demonstrated, SARS-CoV-2 infection requires the ACE-2 receptor to coexist with type II serine protease trans-membranes (TMPRSS2) [25]. TMPRSS2 expression was not evaluated in the present study. However, as recently reviewed by Lazartigues et al., in silico studies indicate that thyroid tissues, with no gender difference, exhibit a high expression of the TMPRSS2 mRNA [35].

The results of the present study suggest that the thyroid might be vulnerable to SARS-CoV-2 infection. This would fit with the recent clinical descriptions of COVID-19-related SAT [17–20]. It is interesting to remember that thyroid repercussions were also described during the 2002 outbreak of SARS-CoV [17, 23]. The expression of ACE-2 was related to the SARS coronavirus aggression in other endocrine glands. In particular, Yang et al. [36] reported that ACE-2 is expressed in the endocrine pancreas, which, after being infected by the SARS coronavirus, undergoes islet damage and impaired insulin release. More recently, Liu et al. [37] showed that some patients with COVID-19 disease experience pancreatic injury leading to insulin deficiency.

In conclusion, the results of the present study clearly indicate that the mRNA encoding for the ACE-2 receptor is expressed in follicular thyroid cells, making them a potential target for SARS-COV-2 entry. Future clinical studies in patients with COVID-19 will be required to improve our understanding of the thyroid repercussions of SARS-CoV-2 infection.
